# 

**Published:** 1890-01

**Authors:** 


					﻿Our number.—Readers will please notice that our number
is changed from 1123 to 11125 Spruce Street. We have not
moved our office. Our number was changed by the city au-
thorities to make room for some new houses near us.
NOTICE.
All articles for publication, books for review, business communications, etc.,
must be sent to Editors, 1125 Spruce Street, Philadelphia.
Subscribers will please notice that this Journal will be sent until arrears
are paid and it is ordered discontinued.
NOTICE TO SUBSCRIBERS.
In order to settle up their accounts, the editors {would he
greatly obliged to receive prompt payment from all sub-
scribers who are in arrears.
The delay in issuing the Repertory number (the combined
issue for October and November) has been unavoidable, and
is greatly regretted by the editors.
				

